# Diagnostic Accuracy of Platelet Count and Platelet Indices in Noninvasive Assessment of Fibrosis in Nonalcoholic Fatty Liver Disease Patients

**DOI:** 10.1155/2017/6070135

**Published:** 2017-12-31

**Authors:** Tamara Milovanovic Alempijevic, Milica Stojkovic Lalosevic, Igor Dumic, Nevena Jocic, Aleksandra Pavlovic Markovic, Sanja Dragasevic, Ivana Jovicic, Snezana Lukic, Dragan Popovic, Tomica Milosavljevic

**Affiliations:** ^1^Clinic of Gastroenterology and Hepatology, Clinical Center of Serbia, 11000 Belgrade, Serbia; ^2^Faculty of Medicine, University of Belgrade, 11000 Belgrade, Serbia; ^3^Mayo Clinic College of Medicine and Sciences, Rochester, MN, USA; ^4^Mayo Clinic Health System, Eau Claire, WI, USA

## Abstract

**Objective:**

Keeping in mind the rising prevalence of nonalcoholic fatty liver disease (NAFLD) and the need to establish noninvasive tests for its detection, the aim of our study was to investigate whether platelet count (PC), mean platelet volume (MPV), and platelet distribution width (PDW) can predict the presence of liver fibrosis in this group of patients.

**Methods:**

In 98 patients with NAFLD and 60 healthy volunteers, complete blood counts with automated differential counts were performed and values of PC, PDW, MPV, and PCT were analyzed.

**Results:**

Patients with NAFLD had lower PC and higher MPV, PCT, and PDW compared to the controls (*P* < 0.05). When NAFLD group was stratified according to severity of liver fibrosis, there was a statistically significant difference in the average values of PDW and PC between the groups (*P* < 0.05).

**Conclusion:**

Patients with NAFLD have significantly higher values of PCT, PDW, and MPV when compared to the healthy controls. Further studies are needed to establish their potential use for prediction of the degree of liver steatosis and fibrosis in NAFLD patients.

## 1. Introduction

Nonalcoholic fatty liver disease (NAFLD) is the leading cause of chronic liver disease worldwide, affecting 20–30% of population in Western countries [1]. NAFLD is a spectrum from simple steatosis with favorable prognosis to nonalcoholic steatohepatitis (NASH), which may progress to cirrhosis and its complications [2]. The hallmark of NAFLD is intrahepatic deposition of triglycerides and the leading factors in this process are insulin resistance and energy misbalance. NAFLD is considered as hepatic manifestation of the metabolic syndrome [3]. Although a liver biopsy is the gold standard for establishing the diagnosis of NAFLD, we must be aware of its invasiveness, patients' discomfort, risk of severe complications, and its high cost. Bearing in mind the rising prevalence of NAFLD and the fact that it has become one of the most common indications for liver transplantation, there is a need to establish noninvasive diagnostic markers for early detection and monitoring of the disease progression [4–6]. Several noninvasive scores for predicting liver fibrosis have already been described: the aspartate aminotransferase to alanine aminotransferase ratio (AAR), the aspartate aminotransferase to platelet ratio index (APRI), fibrosis index (FI), fibrosis-cirrhosis index (FCI), and FIB-4 index (based on age, aspartate and alanine aminotransferase, and platelet counts). Despite the suggestion that these scores correlate with degree of liver fibrosis, there is no sufficient data supporting their everyday use, yet.

Platelets, along their well-known role in hemostasis, are active participants in the process of liver inflammation. They promote leukocyte recruitment through hepatic sinusoids and activate effector cells [7]. Additionally, there are studies suggesting that function and morphology of platelets are altered in patients with diabetes mellitus and metabolic syndrome [8]. MPV, PDW, and PCT (platelet indices) are the indicators of platelet function and activation. It has been suggested that values of platelet indices closely correlate with the presence of insulin resistance and its severity and complications [9, 10].

The aim of our cross-sectional case control study was to evaluate whether platelet count and platelet indices can accurately predict severe steatosis and liver fibrosis in patients with NAFLD patients and to compare their diagnostic accuracy with the other noninvasive scores that have been already published and validated.

## 2. Patients and Methods

A total of 98 patients diagnosed with NAFLD and 60 sex- and age-matched healthy volunteers without NAFLD were included in this prospective study from March to September 2016. All patients were diagnosed with NAFLD based on history, physical examination, laboratory testing, and ultrasound imaging. The diagnosis of NAFLD required the exclusion of secondary causes of liver disease and daily alcohol consumption (≥20 g for men and >10 g for women) [2]. The exclusion criteria were the following: age < 18 years, presence of any other chronic liver disease (CLD), hepatocellular carcinoma, severe chronic extrahepatic disease, hospital admission due to other chronic illnesses, or presence of human immunodeficiency virus infection. All patients provided a written informed consent prior to inclusion in the study.

Data necessary for the diagnosis of the metabolic syndrome [2] were collected from the patients' records. Physical examination of each patient included body weight, height, waist circumference, calculation of body mass index (BMI), and measurement of blood pressure (BP).

Blood samples were collected after 12 hours of fasting. Analyses included PC, platelet indices, liver function tests (AST, ALT, gamma-glutamyl transferase (GGT), and alkaline phosphatase (ALP)), lipid profile, and fasting blood sugar and insulin levels. Analysis of hematological parameters along with platelets and their indices was performed in whole blood anticoagulated with EDTA within 4 hours after collection, using Coulter® LH 750 Hematology Analyzer (Beckman Coulter, USA). Analysis of biochemical parameters was performed using Olympus AU2700 (Olympus Co. Ltd., Tokyo, Japan). For each patient, we calculated homeostasis model assessment-estimated insulin resistance (HOMA-IR) [11].

Sonographic evaluation (US) of hepatic steatosis was performed using five criteria: parenchymal brightness, liver to kidney contrast, deep beam attenuation, bright vessel walls, and gallbladder wall definition [12]. Grading of diffuse hepatic steatosis was used to evaluate the extent of fatty changes in the liver. Grades I to III were defined as follows:

  Grade I: increased hepatic echogenicity with visible periportal and diaphragmatic echogenicity
  Grade II: increased hepatic echogenicity with imperceptible periportal echogenicity, without obscuration of diaphragm
  Grade III: increased hepatic echogenicity with imperceptible periportal echogenicity and obscuration of the diaphragm [13]

For the assessment of steatosis, we used hepatic steatosis index (HSI) and NAFLD liver fat score (NAFLD-LFS) and we used NAFLD fibrosis score (NFS), APRI, FIB-4 index, and BARD score (which takes into account BMI, AAR, and presence of type II diabetes mellitus) for the assessment of fibrosis. All the above scores were calculated using standard formulas on admission (Tables 1 and 2) [11, 12].

We stratified patients with NAFLD into the two groups based on the US findings. Group 1 included patients with mild and moderate steatosis, while group 2 included patients with severe steatosis and possible fibrosis. In the absence of results from a liver biopsy, we used APRI score as validated “gold noninvasive score” to stratify our patients according to the severity of steatosis and fibrosis [11, 12, 14].

A statistical analysis was performed using SPSS 22.0 (SPSS Inc., Chicago, IL, USA). Basic descriptive statistics included the means, standard deviations, ranges, and percentages. Normality of the distribution was examined by the Kolmogorov-Smirnov test. The differences were considered as statistically significant if the two-tailed *P* value was less than 0.05. The sensitivity and specificity as well as the best cut-off value for the platelet indexes were calculated using ROC curves (AUROC).

This study was approved by the Ethics Committee of our institution in keeping with the principles of the Declaration of Helsinki (2000 revision of Edinburgh).

## 3. Results

Clinical, laboratory, and demographic data of patients were summarized in Table 3.

Gender, age, and mean diastolic blood pressure were similar among the NAFLD patients and the control group (*P* > 0.05). NAFLD patients had significantly higher systolic blood pressure, waist circumference, and BMI compared to the controls (*P* < 0.01). Among the biochemical variables, fasting plasma glucose, insulin levels, and triglycerides were significantly higher and high-density lipoprotein was significantly lower in NAFLD group (*P* < 0.01). NAFLD group also had lower PC and higher MPV, PCT, and PDW (*P* < 0.05).

**Figure 1:**
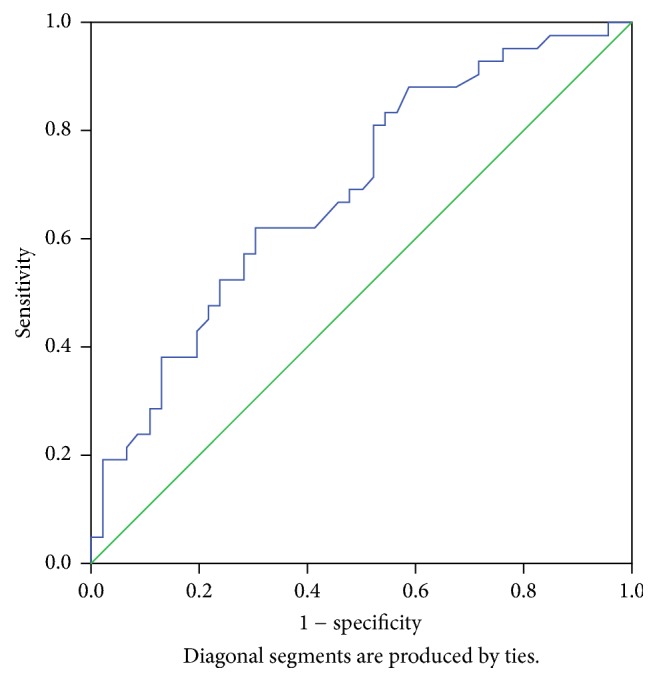
ROC curve 1. Receiver Operating Characteristic (ROC) Curve illustrates sensitivity and specificity of mean PDW values for detection of mild, moderate, and severe steatosis.

When we stratified NAFLD patients into the two groups, we found a statistically significant difference in the average values of PDW and PC between the groups (*P* = 0.04, *P* = 0.03, and *P* < 0.05) with PDW cut-off value of 16.18 for the presence of severe steatosis and possible fibrosis with sensitivity of 88.1% and specificity of 32.6% and AUROC of 0.688 (Figure 1). There were no differences between these groups with regard to MPV and PCT (*P* > 0.05).

When these groups were further stratified according to APRI score (more than or equal to 0.7), we found a statistically significant difference in values of PC, PDW, and PCT between the two groups (*P* = 0.00, *P* = 0.00, *P* = 0.006, and *P* < 0.05) (Table 4).

We found a statistically significant negative correlation between PC and APRI (*P* = 0.00; *r* = −0.530), FIB-4 (*P* = 0.00; *r* = −0.480), and NFS (*P* = 0.00; *r* = −0.320) scores, respectively. Additionally, we found statistically significant negative correlations between PDW and APRI (*P* = 0.00; *r* = −0.5629), FIB-4 (*P* = 0.00; *r* = −0.553), and NFS (*P* = 0.00; *r* = −0.346) scores, respectively.

The results of our study suggest that there is a statistically significant negative correlation between PCT and PDW (*P* = 0.06; *r* = −0.252), as well as a significant positive correlation between PDW and MPV (*P* = 0.04; *r* = 0.261).

## 4. Discussion

The ability to determine the degree of the liver steatosis and fibrosis as well as to predict the progression of disease is essential in the management of patients with NAFLD. A liver biopsy has been used as a gold standard for this purpose over many years. However, its invasive nature, high cost, and risk for development of severe complications (bleeding in particular) resulted in the development of noninvasive tests. These tests consist of different scores derived from various combinations of serologic markers as well as noninvasive imaging modalities [15]. Over the last decade, particularly promising imaging modality has emerged, ultrasound-based transient elastography (TE) with controlled attenuation parameter (CAP). This technique is based on mechanical excitation of the hepatic tissue with monitoring of the resulting tissue response. The fact that fibrotic tissue differs from healthy tissue in the way that it responds to excitation is used to determine level of steatosis and fibrosis. This technique is particularly useful, since it eliminates sampling errors, which was common with liver biopsy, in addition to its noninvasiveness [16, 17]. However, while it is being used more commonly, high cost of the machine is still an obstacle for use in many developing countries. Hence, there is an ongoing interest for discovery of the cheaper and easily available serologic tests for detection of liver steatosis and fibrosis and estimation of the severity grades. Multiple serologic markers reflective of liver function (AST, ALT, bilirubin, PC, GGT, ALP, and haptoglobin) have been used in combination to formulate diagnostic and prognostic scores that would be an alternative to liver biopsy. While some of them are more simple (AAR), some of them include complicated algorithms and multiple variables (FibroTest).

The current research studies suggest that platelets have a role in the process of liver fibrosis by decreasing expression of the principal fibrogenic cytokine TGF-*β* and by increasing expression of matrix metalloproteinases [18, 19]. Subsequently, an inverse correlation occurs between progression of liver fibrosis and platelets. Taking this into account, PC is presently included in many prognostic scores for fibrosis and cirrhosis of the liver. Some previous studies have described that lower PC is related to the more advanced fibrosis; however, only few of these studies assessed PC in NAFLD patients [20]. Unlike platelets, the platelet indices are not widely investigated as the markers of liver steatosis and fibrosis. They might prove to be very useful in the future as a part of diagnostic scores for detection of liver steatosis and fibrosis in patients with NAFLD.

Our study aimed to determine the association between PC and platelet indices with the presence of fibrosis in NAFLD patients and we found an inverse correlation between PC and liver fibrosis, similar to previously published data [18, 19].

Platelet functions can be affected by platelet size, density, other comorbidities, and age. Larger platelets have higher quantity of granules and adhesion receptors, which results in an increase in platelet activation [21]. PDW directly refers to platelet size, changes with platelet activation, and reflects the heterogeneity in platelet morphology [7]. Our study suggests that NAFLD patients have higher values of PDW compared to controls. Study of Cao et al. showed that PC and PDW negatively correlate with the stage of fibrosis, which is in accordance with results that we found in the present study [22].

The results of Ozhan et al. suggest that lower PC and higher MPV are independent predictors of NAFLD [23]. Several independent studies have reported that steatosis was associated with an elevation in MPV [23–27]. A large Korean study has demonstrated a significant association between the presence of NAFLD and higher MPV values in 628 obese volunteers [24]. In our study, the NAFLD group had significantly higher values of MPV compared to the controls, which is similar to the published data.

To the best of our knowledge, there are no studies that investigated potential use of PCT for estimation of the degree of liver steatosis/fibrosis in patients with NAFLD. In the current study, we have not found any significant difference in the values of PCT between NAFLD groups 1 and 2; however, we have found significant difference in the values of PCT between NAFLD patients and the controls. This can be potentially useful as quick and simple parameter for orientation towards patients with suspected NAFLD.

## 5. Conclusion

In conclusion, our study demonstrates that patients with NAFLD have significant increase in the values of PCT, PDW, and MPV. We will need larger studies to investigate potential use of PC and platelet indices and their inclusion in the diagnostic algorithms for noninvasive assessment of degree of steatosis and fibrosis in NAFLD patients. Their use may be beneficial considering that they are simple, easy to measure, and cost-effective and are routinely checked in everyday practice.

## Figures and Tables

**Table 1:** Formulas for calculating noninvasive scores for steatosis.

Noninvasive index of steatosis	Formula
HSI	8*∗*ALT/AST + BMI + 2 if DM + 2 if female
NAFLD-LFS	−2.89 + 1.18*∗*metabolic syndrome (yes = 1, no = 0) + 0.45*∗*type 2 DM (yes = 1, no = 0) + 0.15Io + 0.04*∗*AST − 0.94*∗*AST/ALT

HSI: hepatic steatosis index; NAFLD-LFS: NAFLD liver fat score.

**Table 2:** Formulas for calculating noninvasive scores for fibrosis [12, 13].

Noninvasive index of fibrosis	Formula
NFS	1.675 + 0.037 × age (years) + 0.094 × BMI (kg/m^2^) + 1.13 × IFG/diabetes (yes = 1, no = 0) + 0.99 × AST/ALT ratio − 0.013 × platelet (×10^9^/l) − 0.66 × albumin (g/dl)
APRI	[(AST/upper normal limit AST ) × 100]/platelets (10^9^/L)
FIB-4	(Age × AST)/(platelets × sqr (ALT))
BARD	BMI ≥ 28 = 1p, AST/ALT ratio (AAR) ≥ 0.8 = 2p, DM = 1p

NFS: NAFLD fibrosis score; APRI: AST platelet ratio index; FIB-4: fibrosis-4; BARD: BMI, AST/ALT ratio, and diabetes [12, 13].

**Table 3:** Clinical, laboratory, and demographic data of NAFLD patients compared to controls.

	NAFLD	Control cases	*P* value
Age	51.8 ± 14.6	50.4 ± 14.1	>0.05
Gender: *male*/*female*	56/42	32/28	>0.05
BMI	29.3 ± 4.7	26.2 ± 3.6	<0.01
Waist circumference	109.4 ± 8.9	108.2 ± 6.6	<0.05
Systolic BP	130.3 ± 8.9	124 ± 6.6	<0.01
Diastolic BP	82.6 ± 6.4	81.5 ± 4.4	>0.05
Glucose	6.6 ± 2.04	5.8 ± 1.5	<0.01
Urea	6.5 ± 5.9	6.6 ± 7.5	>0.05
Creatinine	77.5 ± 15.2	73.3 ± 21.1	>0.05
Total cholesterol	5.78 ± 1.1	5.72 ± 1.4	>0.05
LDL cholesterol	3.6 ± 0.9	2.6 ± 1.1	<0.01
HDL cholesterol	1.2 ± 0.6	1.7 ± 0.4	<0.01
Triglyceride	2.4 ± 1.7	1.7 ± 0.9	<0.01
AST	33.1 ± 19.3	22.3 ± 11.2	<0.01
ALT	46.1 ± 25.8	22.1 ± 7.8	<0.01
ALP	72.3 ± 23.0	73 ± 19.2	>0.05
GGT	73.6 ± 82.9	44 ± 12.1	<0.01
WBC	7.02 ± 1.7	7.2 ± 2.6	>0.05
PC	218.4 ± 56.8	255.3 ± 77.9	<0.01
MPV	9.1 ± 1.3	7.6 ± 1.1	<0.01
PDW	16.7 ± 0.7	15.9 ± 0.7	<0.01
PCT	0.2 ± 0.1	0.1 ± 0.0	<0.01

**Table 4:** APRI score according to platelet count and indices.

	APRI ≥ 0.7	APRI < 0.7	*P*
PC (mean ± SD)	154.71 ± 44.92	226.91 ± 52.90	<0.01
MVP (mean ± SD)	9.21 ± 1.05	9.04 ± 1.46	0.581
PCT (mean ± SD)	0.14 ± 0.03	0.22 ± 0.16	<0.01
PDW (mean ± SD)	16.91 ± 0.35	16.55 ± 0.77	<0.01

## Additional Points

*Limitations of the Study.* NAFLD diagnosis was not confirmed by liver biopsy and baseline analyses may not reflect patient's condition over prolonged period of time. Additionally, low AUROC suggest that there is a need for a larger cohort.
